# XDMOM: A Real-Time Moving Object Detection System Based on a Dual-Spectrum Camera

**DOI:** 10.3390/s22103905

**Published:** 2022-05-21

**Authors:** Baoquan Shi, Weichen Gu, Xudong Sun

**Affiliations:** 1 School of Mechano-Electronic Engineering, Xidian University, Xi’an 710071, China; weichen.gu@stu.xidian.edu.cn (W.G.); 17693103117@163.com (X.S.); 2 Shaanxi Key Laboratory of Space Extreme Detection, Xidian University, Xi’an 710071, China; 3 Center of Innovative Industrial Design, Xidian University, Xi’an 710071, China

**Keywords:** moving object detection, video surveillance system, real-time, all-day monitoring, outdoors, in the wild

## Abstract

A low-cost and power-efficient video surveillance system, named XDMOM, is developed for real-time moving object detection outdoors or in the wild. The novel system comprises four parts: imaging subsystem, video processing unit, power supply, and alarm device. The imaging subsystem, which consists of a dual-spectrum camera and rotary platform, can realize 360-degree and all-day monitoring. The video processing unit uses a power-efficient NVIDIA GeForce GT1030 chip as the processor, which ensures the power consumption of the whole system maintains a low level of 60~70 W during work. A portable lithium battery is employed to supply power so that the novel system can be used anywhere. The work principle is also studied in detail. Once videos are recorded, the single-stage neural network YOLOv4-tiny is employed to detect objects in a single frame, and an adaptive weighted moving pipeline filter is developed to remove pseudo-targets in the time domain, thereby reducing false alarms. Experimental results show that the overall correct alarm rate of the novel system could reach 85.17% in the daytime and 81.79% at night when humans are monitored in real outdoor environments. The good performance of the novel system is demonstrated by comparison with state-of-the-art video surveillance systems.

## 1. Introduction

Moving object detection is widely used in intelligent video surveillance, traffic monitoring, pedestrian detection, robot navigation, driver assistance, etc. [1], and many approaches have been proposed in the past. According to whether or not a neural network is involved, existing approaches can be divided into two categories or a combination of them: traditional methods [2,3,4,5,6,7] and neural-network-based methods [8,9,10,11,12]. Traditional methods generally use the characteristics of moving objects in image sequences to detect and identify through various video/image processing algorithms, while neural-network-based methods firstly train a neural network using training datasets, then perform the detection.

### 1.1. Related Works

Classic traditional methods include frame difference [2], background subtraction [3], optical flow [4], etc. The main idea of frame difference is to extract the moving object according to the different positions of the targets in different frames [2]. This type of method is simple and can satisfy real-time requirements. However, the detection results often show cavitation and low detection accuracy, and cannot be directly applied to a moving camera. Background subtraction methods build a background model firstly based on statistical principles, and then compare the frame to be tested with the background model to segment the moving objects. Typical background models include the Gaussian mixed model (GMM) [5], CodeBook [6], and ViBe [7]. Background subtraction methods are simple in calculation, fast in speed, high in accuracy, and good in static scenes, but are sensitive to interference factors such as light, leaf shake, and water fluctuation [1], hence they are not suitable for moving object detection under dynamic scenes. Optical flow methods use the time-varying optical flow characteristics of moving targets to establish the optical flow constraint equation for moving object detection [4]. The advantages are that they can detect moving objects without prior knowledge of the scene, thus being suitable for dynamic scenes. The shortcoming is that the computational burden is heavy.

Neural-network-based methods can be divided into two types: two-stage detection methods [8,9] and single-stage detection methods [10,11,12]. Two-stage detection methods are composed of two steps: generation of candidate regions and classification regression of the detected objects. R-CNN [8] and SPP-Net (spatial pyramid pooling network) [9] are classic two-stage detection methods. These methods need to select candidate regions in images in advance, and then classify and locate the object, hence they cannot easily satisfy real-time detection requirements. Single-stage detection methods extract the candidate regions directly, and then continue the classification regression to the candidate regions. YOLOv1 [10] to YOLOv4 [11] are typical single-stage detection methods. These approaches only need one neural network to predict the object classification and location, and can thus satisfy real-time detection requirements [12].

Although a great number of moving object detection methods have been reported in the literature as mentioned above [1,2,3,4,5,6,7,8,9,10,11,12], very few of them can be directly applied to an actual video surveillance system. The reason is that an actual video surveillance system, especially when used outdoors or in the wild, always equips a low-power processor with limited processing capability for long-term monitoring, therefore most of the moving object detection methods cannot achieve real-time performance [13]. Thus, developing a practical and affordable video surveillance system for real-time moving object detection is meaningful and valuable. The well-known video surveillance system W^4^ was an early attempt to detect and track people in an outdoor environment [14]. In the past decade, some state-of-the-art moving object detection systems have been developed.

Mori et al. [15] presented an FPGA-based omnidirectional vision system based on a background subtraction algorithm for moving object detection in mobile robotic applications. The detection error was about 24% at a distance of 200 cm, thus the system was not suitable for long-distance detection. Wang et al. [16] developed a real-time small moving object detection system based on infrared images. The system uses an FPGA chip and a DSP chip as the main computing elements, and the detection speed can reach 22 fps. Nevertheless, the system is not suitable for all-day conditions, especially when the environmental temperature is high, such as when the sun is shining. Moon et al. [17] implemented an SoC system for real-time moving object detection based on a 32 bit processor ARM922T and an FPGA. The produced SoC system can reach a speed of 15 fps; however, when detecting a moving object, the system has difficulty in preventing the moving object area from reacting sensitively to the illuminance change of an identical object since it detects movement by a difference from the previous image, i.e., the system is sensitive to illuminance changes. Dong et al. [18] designed a moving object tracking system by combining classic object detection and tracking algorithms. Since QiTianM4330 desktop was employed, it was a high-power-consumption system and was not suitable for monitoring moving objects outdoors or in the wild.

Iqbal et al. [19] presented a quadcopter-based solution to monitor desired premises for any unusual activities based on R-CNN. However, images captured by the aerial surveillance system must be transmitted to a workstation on the ground for analysis, which affects the real-time performance. Alam et al. [20] proposed a real-time surveillance system using a low-cost drone (UAV), in which the large computation tasks were moved to the cloud while keeping limited computation on-board the UAV device using edge computing techniques. Since the video streams must be transmitted to the cloud, there would exist end-to-end delay. Angelov et al. [21] designed and implemented a moving object detection system AURORA mounted on a DJI hexacopter S800. The system was able to detect, by on-board processing, any moving objects at a rate of 5 fps while at the same time sending only important data to a control station located on the ground. Rodriguez-Canosa et al. [22] developed a real-time moving object detection and track system DATMO on an onboard UAV computer based on optical flow. Although the camera recorded images at a rate of 30 fps, the moving object detection frequency could only reach 5 to 10 fps. Huang et al. [13] presented a visual-inertial drone system REDBEE that runs on the Snapdragon Flight board for real-time moving object detection. The major shortcoming of aerial surveillance systems based on UAV platforms is that the power consumption of the whole system is very large; for example, the power consumption of a DJI hexacopter S800 is more than 720 W, which results in the drones flying for less than twenty minutes, thus long-term monitoring cannot be achieved.

In summary, the drawbacks of existing video surveillance systems are analyzed from several aspects. Firstly, the time conditions (day or night) are not considered; most of the video surveillance systems cannot work at night [15,17,18,19,20,21,22]. Secondly, some video surveillance systems are only suitable for indoor or close-range monitoring [15,17,18]. Thirdly, some systems have a low detection speed of only about 5~10 fps, though the acquisition rate of their cameras can reach 30 fps or faster [21,22]. Moreover, some of the aerial surveillance systems depend on a workstation on the ground to process video streams, which would result in end-to-end delay [19,20]. Finally, the power consumption of the aerial surveillance systems is very large since the drones are energy-consuming vehicles [13,19,20,21,22], which thereby makes long-term monitoring impossible.

### 1.2. Contribution of Our Work

In this paper, a novel video surveillance system, named XDMOM, is developed for moving object detection outdoors or in the wild. Compared with state-of-the-art video surveillance systems, as shown in Table 1, the advantages of the novel system are summarized as follows:

(1) Power-efficiency. The power consumption of the whole system is about 60~70 W during working, thus it is applicable outdoors or in the wild by using a portable lithium battery as the power supply. Moreover, owing to power-efficiency, the novel system can realize long-term monitoring.
(2) Real-time moving object detection. According to experiments, the novel system can run at a maximum speed of 35 fps. However, the capture rate of the cameras is 25 fps, thereby the novel system works at a speed of 25 fps in practical applications.
(3) All-day monitoring. By means of a dual-spectrum camera, the novel system can work during the day and/or at night.
(4) 360-degree panoramic monitoring without blind spots. Owing to the rotary platform, the novel system realizes 360-degree panoramic monitoring.
(5) High correct alarm rate. An adaptive weighted moving pipeline filter is proposed for pseudo-target removal in the time domain, and experimental results show that the correct alarm rate of the novel system can reach 85.17% during the day and 81.79% at night in real outdoor environments.

## 2. System

In this section, the architecture of the novel video surveillance system XDMOM will be described in detail. Afterwards, the core indicators will be listed.

### 2.1. Composition of XDMOM

**Figure 1:**
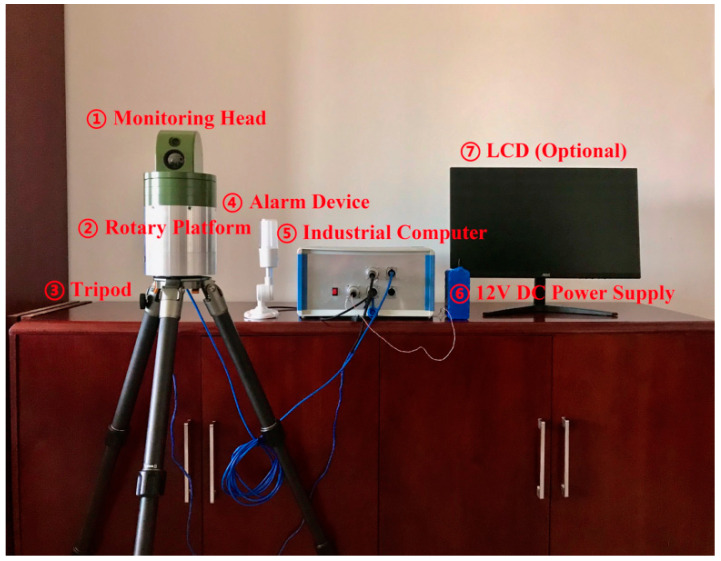
Hardware composition of the novel system. In practical use, the LCD is unnecessary.

**Figure 2:**
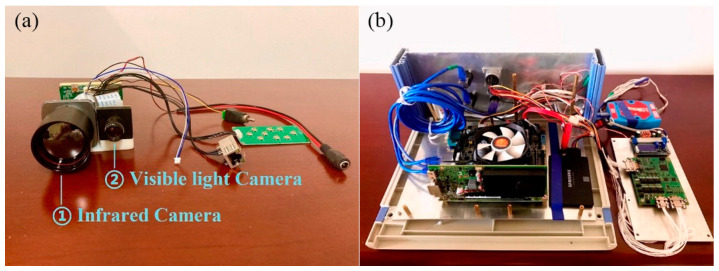
Details of (**a**) dual-spectrum camera (VS and IR camera) and (**b**) industrial computer.

The hardware of XDMOM includes a monitoring head, a rotary platform fixed on a tripod, an industrial computer, a 12 V DC power supply, an alarm device, and an LCD screen (optional), as shown in Figure 1. The monitoring head contains a dual-spectrum camera (Figure 2a), which consists of a visible-spectrum (VS) camera employed for monitoring in the daytime and an infrared (IR) camera employed for monitoring at night. The resolution of the dual-spectrum camera is 720 × 576 and its capturing frame rate is 25 fps. The monitoring head is mounted on the rotary platform, whose rotation speed is within the range 0~45 rpm, and they together form the imaging system of XDMOM. Owing to the dual-spectrum camera and the rotary platform, XDMOM can realize 360-degree and all-day monitoring.

The industrial computer (Figure 2b), which consists of an Intel SKYBAY motherboard (100 Series/C230 Series Chipset Family-A146, Advantech, Suzhou, China), Intel i5-7500T CPU, NVIDIA GeForce GT1030 GPU (2 G memory, 384 CUDA Cores, and 30 W power consumption, the main video processing chip), and Samsung SSD (256 G), is designed to process videos in real-time. Furthermore, the industrial computer connects with other components and controls them to work as expected. Specifically, it controls the rotary platform to rotate at a given speed, and sends signals to the dual-spectrum camera to record videos at day or night. The alarm device communicates with the industrial computer through a USB port, and sends both visual and audible signals to alert users once moving objects are detected. The 12 V DC power supply is a portable lithium battery and can provide power for XDMOM outdoors or in the wild. The LCD screen is an optional device, which is employed to display the monitoring results if necessary.

**Figure 3:**
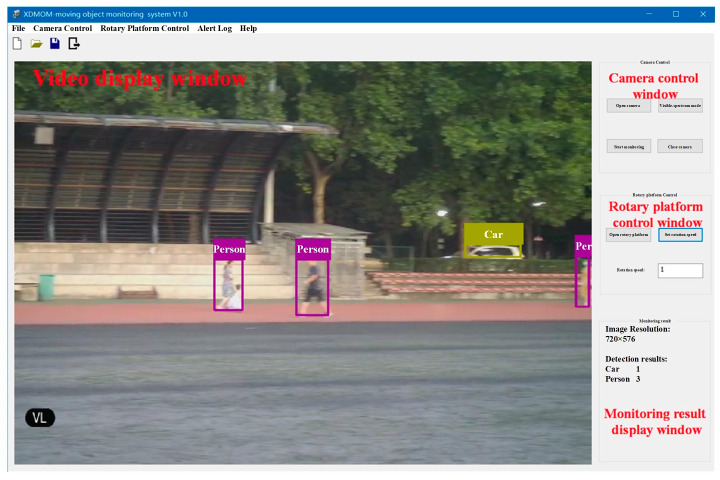
Software of the novel system in visible-spectrum mode.

**Figure 4:**
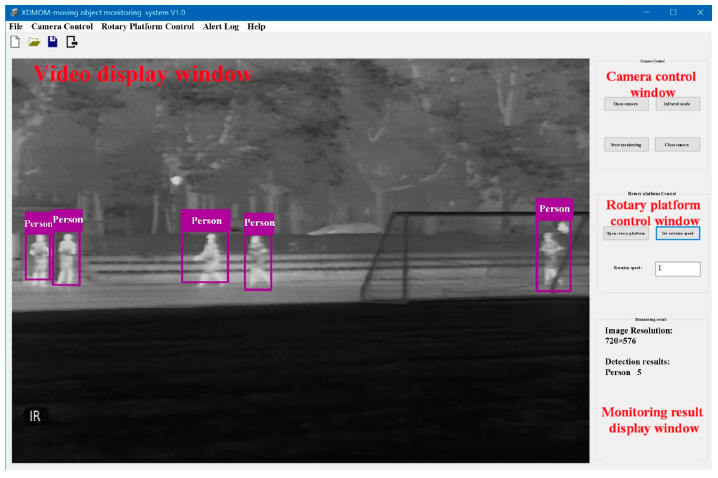
Software of the novel system in infrared mode.

The software of XDMOM includes two monitoring modes: visible-spectrum (VS) mode (Figure 3) and infrared (IR) mode (Figure 4). The VS mode is designed to monitor during the day or under better lighting conditions, and the VS camera will be activated to capture videos in this mode. The IR mode is designed to monitor at night or under poor lighting conditions, and the IR camera will be activated to capture videos in this mode. There are mainly four windows: video display window, camera control window, rotary platform control window, and monitoring result display window. In general, the software of XDMOM runs in the background, and displays only when moving objects are monitored. The video processing program, which mainly includes moving object detection in a single frame in the space domain and pseudo-target removal in the time domain, is integrated in the software, and primarily runs on an NVIDIA GeForce GT1030 GPU.

### 2.2. Core Indicator of XDMOM

The core indicators of XDMOM are listed in Table 2. The system can realize real-time, 360-degree, and all-day monitoring by means of the dual-spectrum camera, rotary platform, and NVIDIA GeForce GT1030 GPU. The monitoring range is up to 100 m. The power consumption of the whole system is about 60~70 W, thus the system is able to work continuously for more than 30 h outdoors or in the wild by using a portable lithium battery, whose capacity is 180,000 mAh, as the power supply. The correct alarm rate of the system can reach 85.17% during the day and 81.79% at night when humans are monitored, according to the experimental results. Moreover, the price of the video processing chip NVIDIA GeForce GT1030 GPU is only tens of dollars, and the other components involved can also be found in electronics markets at affordable prices, hence the cost of the novel system is low.

## 3. Work Principle

**Figure 5:**
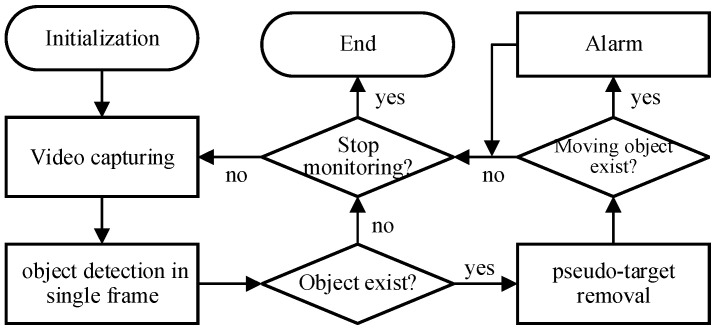
Workflow of the novel system.

The workflow of XDMOM is shown in Figure 5, including initialization, video capturing, object detection in a single frame in the space domain, pseudo-target removal in the time domain, and alarm. The aim of initialization is reading script/configuration files to initialize the necessary system parameters such as the rotation rate of the rotary platform and weights for the neural network employed during the object detection stage. Afterwards, videos are captured continuously until the ‘stop monitoring’ command is received by the system. Whether the VS camera or the IR camera is activated to record videos is determined by users according to the lighting conditions. There is a hardware switch designed for users switching between the VS camera and the IR camera. There is also a soft button in the software to switch the monitoring mode. However, both require human involvement. If a photosensor is integrated in the system in future, automatic switching would thus be achieved.

### 3.1. Moving Object Detection in a Single Frame in the Space Domain

As mentioned before, a number of methods [1] have been proposed to detect moving objects in video/images. However, some traditional object detection methods, such as frame difference methods [2] and background subtraction methods [3], no longer fit the demands of the novel system. The main reason is that the cameras rotate with the rotary platform, i.e., the scene is dynamic; thus, the adjacent frames should be aligned in advance. However, there are fewer feature points/corners in infrared frames, hence it is difficult to match neighboring frames for conducting either frame difference or background subtraction. Moreover, optical flow methods [4] and two-stage neural-network-based methods [8,9] cannot meet the real-time requirements. Therefore, single-stage neural-network-based methods [10,11] are employed.

**Figure 6:**
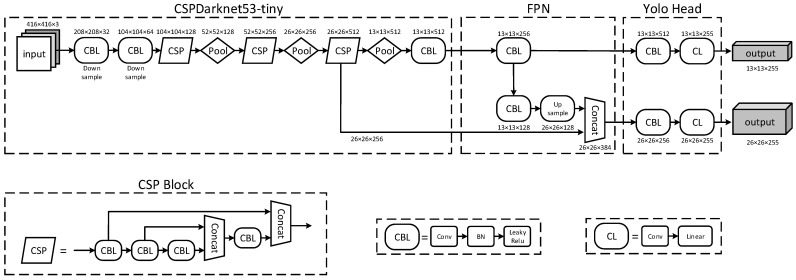
Detailed structure of YOLOv4-tiny.

Among existing single-stage neural-network-based methods, YOLO [10,11] appears to perform well in both detection precision and detection speed [12]. The latest version of YOLO is YOLOv4 [11], which is an upgraded version of YOLOv3. YOLOv4 combines Darknet53 with cross-stage partial (CSP) connections as the backbone for feature extraction, uses spatial pyramid pooling (SPP) as the additional module of CSPDarknet53 for increasing the receptive field, and uses a path-aggregation network (PANet) for parameter aggregation. YOLOv4 also has a lightweight version named YOLOv4-tiny, which reduces the size of network and corresponding parameter numbers to achieve much faster speeds at the expense of accuracy. The structure of YOLOv4-tiny is shown in Figure 6. CSPDarknet53-tiny is applied in YOLOv4-tiny as the backbone network, mainly consisting of three units: (1) CBL module, which contains a convolutional layer, batch normalization layer, and LeakyReLu activation function, is adopted as the basic convolution unit. (2) A CSP block is used as the residual unit to integrate shallow and deep features, which can improve the learning ability of the CNN. (3) Maximum pooling is used as the down-sampling unit, which can enhance the salient features well while simultaneously reducing the number of feature parameters. In addition, feature pyramid networks (FPNs) are used to replace SPP and PANet for speeding up the process of feature fusion. Two-scale feature predictions (26 × 26 and 13 × 13) are performed by YOLOv4-tiny to obtain the detection results. According to experiments, the detection speed of YOLOv4 executed on an NVIDIA GT1030 GPU is about 7 fps, while for YOLOv4-tiny it is 35 fps. Because the frame rate of the dual-spectrum cameras is 25 fps, YOLOv4 cannot meet real-time requirements, so YOLOv4-tiny is adopted here.

### 3.2. Pseudo-Target Removal in the Time Domain

The detection accuracy of YOLOv4-tiny is about 40.2% (AP50) [23]. That is, some moving objects detected by YOLOv4-tiny would be pseudo-targets. Hence, it is necessary to remove pseudo-targets, thereby reducing false alarms.

**Figure 7:**
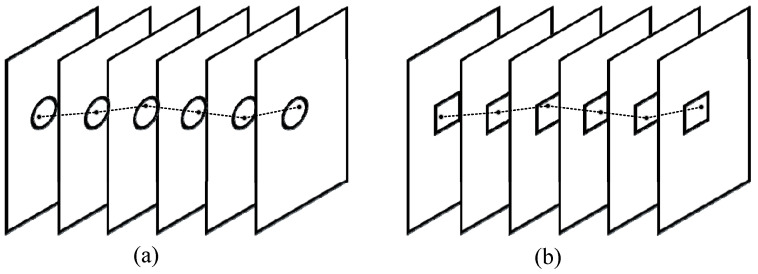
Two classical pipelines: (**a**) circular pipeline and (**b**) rectangular pipeline.

Pipeline filter (PF) [24] is a time-domain filter, which can effectively separate target trajectory from background noise based on the theory of spatial continuity of the target trajectory and incoherence of random noise in the time domain. As shown in Figure 7, there are two common pipelines: circular and rectangular pipelines. Generally, the pipeline is established by the target center *c*, and the size (width *w* and height *h*) and length *N* of the pipeline. The size of the pipeline represents the influence scope in the space domain, while the length of the pipeline represents the number of frames in one pipeline period. Given a threshold *T* (*T* ≤ *N*) specified by users in advance, if the trajectory of objects appears in one pipeline period more than *T* frames, there exists a target in the current pipeline, and vice versa.

**Figure 8:**
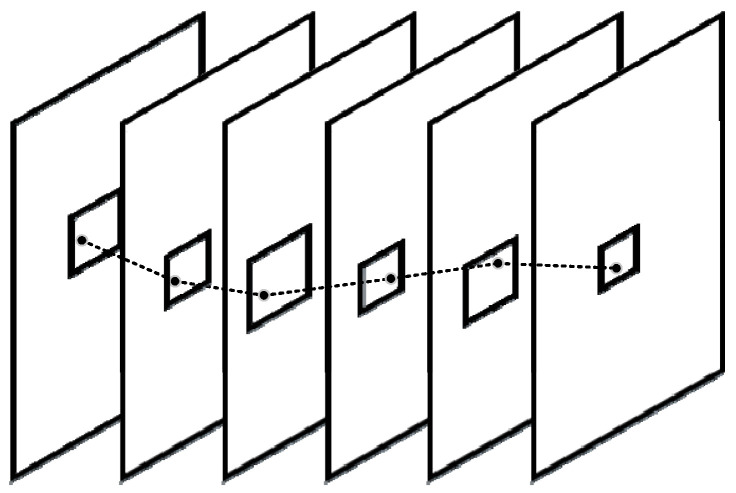
Schematic diagram of the adaptive weighted moving pipeline filtering (AWMPF).

The PF algorithm is only applicable for objects moving at a constant speed in static scenes since the center and size of the pipeline remains the same during one pipeline period. However, the cameras of XDMOM rotate continuously, and the objects in the scene may move at different speeds, therefore an adaptive weighted moving pipeline filter (AWMPF), which is an improvement of the PF algorithm, is proposed. The center and size of the pipeline are thereby calculated as follows:

(1) $$\{\begin{array}{c} w=\alpha \times w_{b}+r_{c}\\ h=\alpha \times h_{b}\\ c_{x}=c_{bx}+0.5\times r_{c}\\ c_{y}=c_{by}\\ r_{c}=1.6\times s \end{array}$$

where $\left(w_{b},h_{b}\right)$ are the width and height of the bounding box of the object detected in each frame, $\left(c_{x},c_{y}\right)$ are the coordinate components of target center *c*, $\left(c_{bx},c_{by}\right)$ are the coordinate components of the bounding box center of the moving object detected in each frame, $r_{c}$ is the compensation of camera rotation, *s* is the rotation speed of the rotary platform, and α is a weight factor. Because the center and size of the bounding box of a moving object detected in each frame change in real-time, the center and size of the pipeline change accordingly as shown in Figure 8.

**Figure 9:**
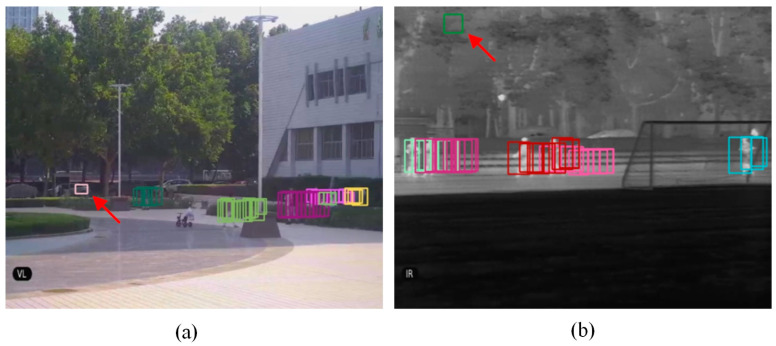
AWMPF applied to (**a**) VS frames and (**b**) IR frames. The rectangles with the same color represent the pipeline of a person. The red arrows are pointing to pseudo-targets.

Owing to the time-varying property, AWMPF can adapt well to the novel system. Figure 9 shows an example, where correctly detected people would be well maintained since their trajectory appears in one pipeline period more than the threshold *T*, while pseudo-targets (to which the red arrows are pointing) will be removed accordingly.

Table 3 shows the comparison of the detection results of a VS video and an IR video with and without applying AWMPF. It can be seen that the false alarms are very large without applying AWMPF; with regard to video 1, the false alarm rate was about 22.22%, which was unacceptable for actual video surveillance. After applying AWMPF, the false alarm rate was reduced to about 1.23%.

### 3.3. Alarm

Once a moving object is confirmed by AWMPF, a signal will be sent to the alarm device to trigger the alarm. Meanwhile, the frames containing moving objects will be stored locally and/or sent to a remote display device.

### 3.4. Evaluation of the System

As mentioned before, the aim of the novel system is to trigger an alarm when detecting a moving object during work. In order to evaluate the performance of the novel system in real applications, several factors, including correct alarm rate, false alarm rate, and missed alarm rate, were defined. A correct alarm was defined as a correct alarm signal sent by the alarm device when a moving object was detected correctly, a false alarm was defined as a false signal sent by the alarm device when a false moving object was detected, and a missed alarm was defined as an alarm signal that should have been sent but was not sent by the alarm device. It can be derived accordingly that the total alarms (or groundtruth) that should be sent by the alarm device during work are the sum of correct alarms and missed alarms. Thus, the correct alarm rate was computed as the correct alarms divided by the total alarms, the false alarm rate was calculated as the false alarms divided by the total alarms, and the missed alarm rate was defined as the missed alarms divided by the total alarms. Ideally, the correct alarm rate should be as large as possible, while the false alarm rate and the missed alarm rate should be as small as possible.

## 4. Results and Discussion

**Figure 10:**
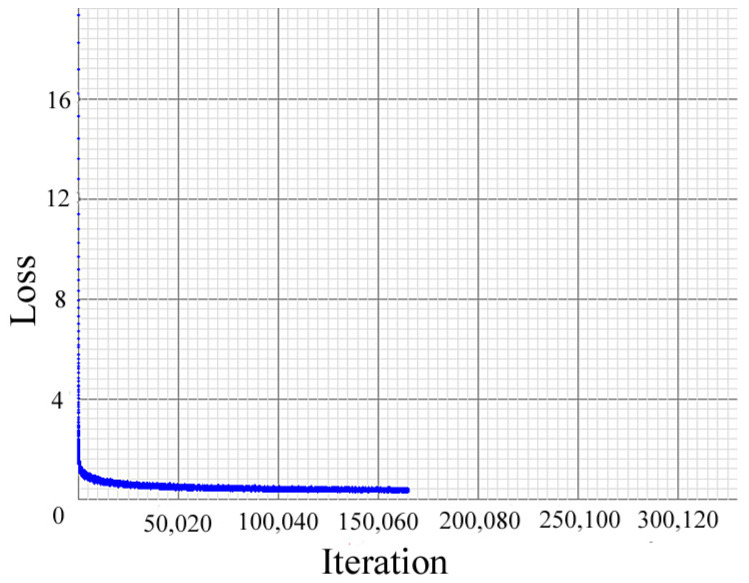
The relationship between loss and iteration during training.

Before the experiments, the neural network was retrained based on the MS COCO dataset [25] and KAIST dataset [26], since XDMOM aims to monitor moving objects. As shown in Table 4, currently, moving objects for retraining include three types: vehicles (car, bus, and truck), humans, and animals (cat, dog, horse, bird, sheep, and cow). In IR mode, only humans were retrained based on the KAIST dataset. Figure 10 shows the relationship between loss and iterations during training, it can be seen that after about 150,000 iterations, the loss was small and almost stopped falling.

**Figure 11:**
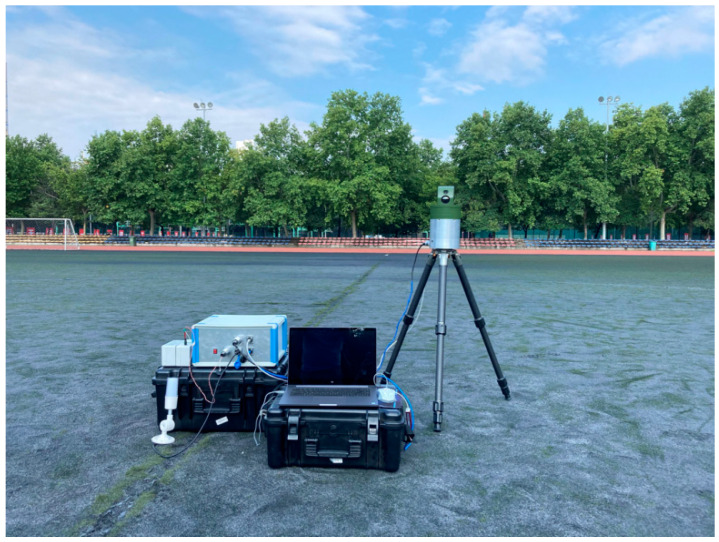
The novel system in the testing scenario.

Experiments were carried out at the school yard of Xidian University as shown in Figure 11. During experiments, a laptop was employed as the display device, and only humans were monitored since there were no moving vehicles or animals in the scene. Finally, five VS videos and four IR videos were captured and processed, and the videos were also saved locally for evaluation purposes. In the experiments, the length of the pipeline was set to 10 (i.e., *N* = 10), the weight factor was set to 0.7 (i.e., α=0.7), and the threshold *T* was set to 5 (i.e., *T* = 5).

**Figure 12:**
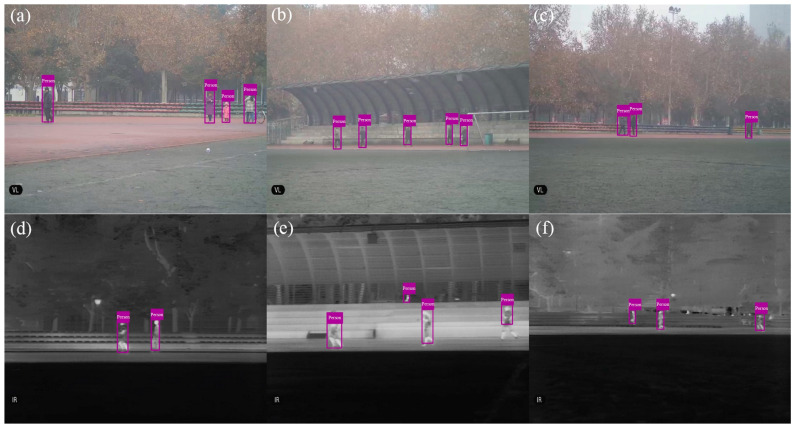
Some screenshots of (**a**–**c**) VS videos and (**d**–**f**) IR videos. Detected moving objects (people) are labeled.

Some screenshots of the videos during the experiments are shown in Figure 12. The experimental results for the VS videos are listed in Table 5. It can be seen that the correct alarm rates are between 82.5% and 87.37%, the false alarm rates are equal to or less than 1.2%, and the missed alarm rates are between 12.63% and 17.95%. The reason why the false alarm rate is extremely low is that some false detection obtained in single frames was removed by the AWMPF.

The experimental results for IR videos are listed in Table 6. It can be seen that the correct alarm rates (overall value is 81.79%) are lower than the monitoring results for VS videos (85.17%), while the false alarm rates (overall value is 5.18%) and missed alarm rates (overall value is 18.21%) are both higher than the monitoring results for VS videos (overall values are 0.34% and 14.83, respectively). The possible reason is that the moving objects have fewer features in IR videos than in VS videos, thereby they are more difficult to identify.

In order to evaluate the processing efficiency of the novel system, four videos, including two VS videos and two IR videos, were processed in an offline manner. As shown in Table 7, the novel system can run at a speed of 35 fps. That is, the novel system can run at a maximum speed of 35 fps, although it works at a speed of 25 fps (the capture rate of cameras is 25 fps) in practical applications.

The power consumption of the novel system was also analyzed according to the statistics of the working voltage and current. As shown in Table 8, the working voltage of the novel system was fixed at 12 V (DC) and the working current fluctuated in the range 5.0 A to 5.8 A (measured using a current monitor); thus, the power consumption of the novel system was about 60~70 W. The power consumption of the novel system is efficient compared with some of the existing moving object detection systems. As can be seen from Table 8, the power consumption of the novel system is about one third of the equipment presented by Dong et al. [18], one sixth of the equipment developed by Alam et al. [20], and one twelfth of the system AURORA [21]. In practical applications, the system is able to work continuously for more than 30 h outdoors or in the wild by using a portable lithium battery whose capacity is 180,000 mAh.

As mentioned before, although there are many algorithms that would obtain high detection precision, most of them cannot be released on an actual video surveillance system because of the limited processing capability of the processors. Among the existing low-cost and power-efficient video surveillance systems, as shown in Table 9, our system performs well. The detection precision of our system is the correct alarm rate. The precisions of other systems were obtained from corresponding papers.

## 5. Conclusions

In the literature, there are many theoretical reports about how to detect moving objects in videos or continuous image frames. However, there are few papers about developing practical and affordable systems. In this study, a low-cost and power-efficient video surveillance system was developed for moving object detection outdoors or in the wild. The architecture and work principles of the novel system were presented in detail. The novel system has such features as real-time, 360-degree, and all-day monitoring, as well as being low-cost and power-efficient. Experimental results show that the overall correct alarm rate of the novel system can reach 85.17% in the daytime and 81.79% at night when humans are monitored in outdoor environments. We hope our work will be helpful in order for others to build their own affordable moving object detection system.

## Figures and Tables

**Table 1:** Performance analysis of the state-of-the-art video surveillance systems.

System	Resolution	Speed/fps	Power-Efficiency	All-Day Monitoring	Long-Term Monitoring
REDBEE [13]	640 × 480	16.2	×	×	×
Mori et al. [15]	800 × 480	26.6	√	×	√
Wang et al. [16]	720 × 576	22	√	×	√
Soc system [17]	640 × 480	15	√	×	√
Dong et al. [18]	640 × 480	25	×	×	√
Iqbal et al. [19]	800 × 600	15.1	×	×	×
Alam et al. [20]	1280 × 720	30	×	×	×
AURORA [21]	640 × 480	5	×	×	×
DATMO [22]	640 × 480	10	×	×	×
**XDMOM**	**720 × 576**	**25**	**√**	**√**	**√**

**Table 2:** Core indicators of the novel system.

Item	Value
Resolution	720 × 576
Frame rate	25 fps
Field of view	360-degree
Monitoring range	100 m
Monitoring interval	All-day monitoring
Power consumption	60~70 W
Correct alarm rate	85.17% during the day and 81.79% at night
Application scene	Outdoors or in the wild

**Table 3:** Comparison of moving object detection results with and without applying AWMPF.

Videos	Total Alarms	False Alarms	
		Without Applying AWMPFM	With Applying AWMPFM
VS	81	18 (22.22%)	1 (1.23%)
IR	139	19 (13.67%)	8 (5.76%)

**Table 4:** Moving objects retrained in the novel system.

Object Type	VS Mode	IR Mode
Vehicles	car, bus, truck	
Humans	person	person/people
Animals	cat, dog, horse, bird, sheep, cow	

**Table 5:** Statistics of monitoring results of VS videos.

Videos	Total Alarms	Correct Alarms	False Alarms	Missed Alarms
1	38	32 (84.21%)	0 (0.0%)	6 (15.79%)
2	95	83 (87.37%)	0 (0.0%)	12 (12.63%)
3	39	32 (82.05%)	0 (0.0%)	7 (17.95%)
4	37	32 (86.49%)	0 (0.0%)	5 (13.51%)
5	81	68 (83.95%)	1 (1.23%)	13 (16.05%)
total	290	247 (85.17%)	1 (0.34%)	43 (14.83%)

**Table 6:** Statistics of monitoring results of IR videos.

Videos	Total Alarms	Correct Alarms	False Alarms	Missed Alarms
1	139	109 (78.42%)	8 (5.76%)	30 (21.58%)
2	143	110 (76.92%)	5 (3.50%)	33 (23.08%)
3	104	91 (87.50%)	6 (5.77%)	13 (12.50%)
4	174	148 (85.06%)	10 (5.75%)	26 (14.94%)
total	560	458 (81.79%)	29 (5.18%)	102 (18.21%)

**Table 7:** The processing time analysis with the novel system running offline. (Note that the system works online at a frame rate of 25 fps).

Videos	Number of Frames	Processing Time (s)	Frame Rate (fps)
VS1	2475	70.714	35
VS2	4775	136.429	35
IR1	2350	67.143	35
IR2	2500	71.429	35

**Table 8:** Power consumption analysis of the novel system.

System	Voltage (V)	Current (A)	Power (W)
XDMOM	12	5.0~5.8	60~69.6
Dong et al. [18]	-	-	>180
Alam et al. [20]	-	-	>400
AURORA [21]	-	-	>720

The values listed in the table for the equipment presented by Dong et al. [18], for the equipment developed by Alam et al. [20], and for the system AURORA [21], comprise only the power consumption of key components; the whole system consumes more power.

**Table 9:** Detection precision comparison of our system with state-of-the-art systems.

Systems	VS Videos	IR Videos
Iqbal et al. [19]	79.0%	--
Alam et al. [20]	83.19%	--
Wang et al. [27]	--	66.0%
Zhang et al. [28]	79.0%	79.68%
**XDMOM**	**85.17%**	**81.79%**

## Data Availability

The data presented in this study are available from the corresponding authors upon reasonable request.
